# Identification of genes associated with disulfidptosis in the subacute phase of spinal cord injury and analysis of potential therapeutic targets

**DOI:** 10.3389/fimmu.2025.1642757

**Published:** 2025-10-20

**Authors:** Shenglong Wang, Xiaochen Su, Wenting Xu, Yonghui Zhao, Yulong Zhang, Yingang Zhang

**Affiliations:** ^1^ Department of Orthopedics, The First Affiliated Hospital of Xi’an Jiaotong University, Xi’an, China; ^2^ The Second Affiliated Hospital of Xi’an Jiaotong University, Xi’an, China

**Keywords:** disulfidptosis, bioinformatics analysis, spinal cord injury, machine learning, molecular docking simulation

## Abstract

**Introduction:**

Disulfidptosis, a recently identified form of regulated cell death, plays a potential role in secondary injury following spinal cord injury (SCI). However, its regulatory mechanisms and therapeutic targets during the subacute phase remain unclear. This study aimed to systematically identify core disulfidptosis-related genes (DRGs) in subacute SCI and explore potential diagnostic biomarkers and therapeutic compounds.

**Methods:**

Two GEO datasets—GSE151371 and GSE45006 were analyzed. Differentially expressed genes (DEGs) were intersected with known DRGs to obtain disulfidptosis-related DEGs (DE-DRGs). Immune cell infiltration was assessed using CIBERSORT, while small-molecule prediction and molecular docking identified candidate compounds. Key diagnostic genes were screened by random forest and LASSO algorithms and validated via receiver operating characteristic (ROC) analysis. A protein–protein interaction (PPI) network was constructed, and Mfuzz clustering analyzed temporal expression patterns. Immunofluorescence staining of rat SCI sections validated IQGAP1 expression and cellular localization by co-labeling with cell-type markers and quantifying co-localization coefficients.

**Results:**

A total of 6,948 DEGs were obtained, among which 8 overlapped with known DRGs. IQGAP1 was significantly upregulated in SCI samples, positively correlated with neutrophil infiltration, and located at the core of the PPI network. It was identified as a key diagnostic gene by both machine learning algorithms, showing a high diagnostic accuracy (AUC = 0.974). Molecular docking indicated a strong binding affinity between IQGAP1 and small molecules such as vitamin E (binding energy < –7.0 kcal/mol). Time-series clustering revealed sustained upregulation of IQGAP1 from day 7 onward in the subacute phase. Functional enrichment analyses (GO, KEGG, GSVA, and GSEA) implicated IQGAP1 in cytoskeleton remodeling, immune regulation, and metabolic reprogramming. Immunofluorescence in SCI rat models confirmed consistent spatial expression patterns.

**Discussion:**

IQGAP1 was identified as a central regulator of disulfidptosis during the subacute phase of SCI, exhibiting strong diagnostic and therapeutic potential. Its association with immune infiltration and metabolic remodeling suggests that targeting IQGAP1 may offer novel strategies for mitigating secondary injury in SCI.

## 1 Introduction

Spinal cord injury (SCI) is a severe neurological disorder that leads to temporary or permanent changes in spinal cord function. It has a high incidence and disability rates, significant economic burden, and often affects younger individuals (1–3).

The global incidence of SCI ranges from 250,000 to 500,000 cases annually, with approximately 17,000 new cases annually in the United States and 66,374 new traumatic cases in China each year (1, 2), posing a significant threat to public health. Traumatic SCI occurs in two stages: primary and secondary injury. Primary injury results in immediate mechanical disruption and dislocation of the spinal column, leading to spinal cord compression or transection (4, 5), and rapidly initiates a cascade of secondary injury events. Secondary injury occurs in acute, subacute, and chronic phases, and is characterized by vascular damage, ionic imbalance, excitotoxicity, free radical production, increased calcium influx, lipid peroxidation, inflammation, edema, necrosis, neuronal apoptosis, axonal demyelination, Wallerian degeneration, axonal remodeling, and glial scarring (2, 6). Secondary injury represents a critical therapeutic target and a key regulatory phase in the treatment of SCI (1). Current clinical interventions mainly include hemodynamic monitoring, early surgical decompression, and management of complications (4, 7), but specific targeted therapies for secondary injury are still lacking.

Evidence suggests that programmed cell death, including apoptosis (8), necroptosis, autophagy, ferroptosis (9), pyroptosis (10), and parthanatos, is widely involved in the occurrence and development of SCI, and may play a crucial role in secondary injury, leading to neurological deficits (11, 12). However, these forms of programmed cell death still fail to comprehensively explain the complex pathological processes. Disulfidptosis, a recently proposed form of programmed cell death (13), is closely related to the accumulation of abnormal disulfide bonds within the cell. When accompanied by glucose deficiency or NADPH depletion, oxidative stress rapidly accumulates inside the cell, ultimately leading to cell death by causing the collapse of the actin filaments (F-actin) network (14). Evidence has shown that disulfidptosis is closely related to the occurrence and development of tumors and has significant implications and value in cancer treatment (13, 15). Emerging evidence suggests a close association between disulfidptosis and central nervous system (CNS) disorders. Oxidative stress is a proposed pathogenic mechanism in CNS diseases, and disulfide stress represents a specific subtype of oxidative stress. Both proteins associated with disulfidptosis and related metabolic pathways have been shown to be significantly linked to CNS disorders (16). Oxidative stress also plays a pivotal role in the secondary injury process of SCI (7, 17), indicating that disulfidptosis may contribute to SCI pathogenesis. Previous studies have found that disulfidptosis-related genes may be involved in the regulation of acute SCI (18, 19), but systematic studies and further validation are still lacking.

Based on this, this study employs various integrated bioinformatics tools to identify key genes closely related to disulfidptosis in SCI. It combines protein-protein interaction (PPI) network, functional enrichment (GO/KEGG), GSVA, immune infiltration analysis, and drug target prediction, and further investigates the potential molecular mechanisms and intervention value by establishing animal models and validating findings through immunofluorescence staining. The results are expected to provide theoretical support for unveiling the molecular mechanisms of disulfidptosis in SCI and offer new candidate targets for precision therapeutic strategies.

## 2 Materials and methods

### 2.1 Dataset preparation

The Gene Expression Omnibus (GEO, http://www.ncbi.nlm.nih.gov/geo/) is an open-access database containing gene chip and high-throughput sequencing datasets. The GSE151371 and GSE45006 expression profile datasets were downloaded from GEO. The GSE151371 dataset includes samples from 10 healthy individuals with no central nervous system (CNS) injury, 10 patients without CNS injury, and 38 individuals with traumatic spinal cord injury (SCI). High-throughput sequencing was performed using the Illumina HiSeq 4000 platform to extract RNA sequences. For analysis, samples were divided into two groups: the control group, consisting of 10 healthy individuals, and the SCI group, consisting of 38 individuals with traumatic SCI. The GSE45006 dataset was based on the GPL1355 platform. The GSE45006 dataset, based on the GPL1355 platform, was generated from a rat SCI model established using the aneurysm clip compression method. The SCI group was divided into three subgroups according to the time points: 1 week, 2 weeks, and 8 weeks post-injury.

### 2.2 Differential expression analysis and selection of differentially expressed disulfidptosis-related genes

Differential expression analysis of the expression profile data from the GSE151371 dataset was performed using the edgeR package in R (version 4.3.1) to identify differentially expressed genes (DEGs) between the healthy control group and the SCI patient group( (20)). The filtering criterion was set to an adjusted *P*-value < 0.05. The DEGs were then intersected with the 24 previously reported disulfidptosis-related genes (DRGs) (13) to obtain the differential expression of DE-DRGs. Additionally, principal component analysis (PCA) was conducted using the stats package, and a PCA scatter plot was generated using the ggplot2 package to assess clustering trends between samples. Venn diagrams, volcano plots, and heatmaps were created using the ggplot2 and ggrepel packages to visually present the expression patterns and distribution characteristics of the DE-DRGs.

### 2.3 Immune infiltration analysis

Immune infiltration analysis was performed using the R package “IOBR”, and the CIBERSORT algorithm was applied to explore differences in immune cell infiltration between different groups. Spearman’s rank correlation coefficient was used to assess the relationship between the expression of diagnostic biomarkers and immune cell infiltration.

### 2.4 Identification of potential compounds and drugs

DSigDB is a database that stores and shares gene expression profiles related to drugs, enabling drug mechanism research and prediction of drug side effects (21). The DSigDB database was accessed through the Enrichr platform (https://amp.pharm.mssm.edu/Enrichr/), where potential drugs and compounds were selected based on their significance (*P*-value), and the top ten candidates were listed.

### 2.5 Molecular docking

The most important gene identified through machine learning, PPI network analysis, and validation using the GSE45006 gene set was selected for molecular docking. The protein corresponding to this gene, along with the top 10 drugs or compounds ranked based on their regulatory effects on DE-DRGs, were selected for molecular docking validation. The 3D structures of these 10 drugs and compounds were obtained from the PubChem database and saved in SDF format. The protein structure of IQGAP1 was retrieved from the PDB database (https://www.rcsb.org/) using the human species model and saved in PDB format. Molecular docking simulations, validation, and visualization were performed using the online CB-Dock2 database (https://cadd.labshare.cn/cb-dock2/index.php) (22, 23). CB-Dock2 is a novel blind docking tool that predicts binding regions for a given protein, calculates the center and size using a curvature-based cavity detection method, and integrates with the docking software AutoDock Vina (1.2.0) to enhance docking accuracy. Additionally, CB-Dock2 ranks binding modes based on Vina scores (kcal/mol) and provides interactive 3D visualization of the binding modes.

### 2.6 Machine learning

In this study, least absolute shrinkage and selection operator (LASSO) regression analysis was used to initially screen candidate disulfidptosis-related biomarkers involved in the pathological process of SCI. The LASSO algorithm was implemented using the “glmnet” R package, which effectively prevents overfitting and performs variable selection and dimensionality reduction. Subsequently, the Random Forest (RF) algorithm was employed to further refine the selection and ranking of biomarkers. RF analysis was conducted using the “randomForest” R package, and feature importance (MeanDecreaseGini) was used to validate and rank the biomarkers identified in the preliminary selection, aiming to identify the key genes with the most diagnostic and predictive value.

Additionally, receiver operating characteristic (ROC) curve analysis was performed, and the area under the curve (AUC) was calculated to assess the sensitivity and specificity of the final selected candidate biomarkers for SCI diagnosis. This analysis helped determine their diagnostic performance in distinguishing between the disease and control groups.

### 2.7 PPI analysis

STRING (https://cn.string-db.org/) is a comprehensive functional protein-protein interaction database and online analysis tool that helps understand protein interactions, pathways, and functional enrichment. In this study, STRING (version 12.0) was used to explore potential interactions between DE-DRGs with a medium confidence score > 0.4. The analysis results were then imported into Cytoscape (version 3.7.2) to construct a visual PPI network. The core genes in the PPI network were identified using the cytoHubba plugin.

### 2.8 Mfuzz clustering analysis

To identify the dynamic expression patterns of genes during SCI, we performed clustering analysis of the time-series gene expression features in the GSE45006 dataset using the Mfuzz package in R. Mfuzz, based on the fuzzy c-means algorithm, effectively captures continuous dynamic changes in gene expression. Through this analysis, we identified key gene clusters with similar expression trajectories at different stages of SCI, which may reflect the biological characteristics of disease progression, tissue damage, and repair processes. Genes with significant and consistent expression trends were then considered as candidate factors closely associated with SCI for further functional analysis and the construction of therapeutic response prediction models.

### 2.9 Gene ontology and Kyoto encyclopedia of genes and genomes pathway enrichment analysis

To elucidate the potential biological functions and signaling pathways of SCI-related genes, we performed GO functional enrichment and KEGG pathway enrichment analyses on DEGs using the clusterProfiler package (version 4.0.5) in R. The significance threshold for both GO and KEGG analyses was set to P < 0.05 to identify biologically significant functions and pathways, providing further insights into the key regulatory mechanisms in the pathological process of SCI.

### 2.10 Gene set enrichment analysis and gene set variation analysis

To further explore the potential pathway activity differences associated with SCI-related genes, we performed GSEA using the clusterProfiler package in R. In the GSEA analysis, a threshold of P < 0.05 and FDR (Q-value) < 0.25 was used to identify significantly enriched pathways, and the gseaplot package in R was employed for visualization of the enrichment results. Additionally, we used the GSVA package to evaluate pathway activity differences between samples, revealing the biological differences at the signaling pathway level among different SCI groups. This analysis provides a biological basis for further research on disease mechanisms and therapeutic strategy exploration.

### 2.11 Animals and SCI model

Female Sprague-Dawley (SD) rats (220–240 g) were purchased from the Animal Experimental Center of Xi’an Jiaotong University. All rats were housed in a controlled environment with appropriate temperature and humidity, with ad libitum access to water and food. This study was approved by the Ethics Committee of the School of Medicine, Xi’an Jiaotong University (Approval No: XJTUAE2024-05).

The rats were weighed and anesthetized with intraperitoneal injection of 2% pentobarbital (30 mg/kg). Under sterile conditions, a T9-T10 laminectomy was performed to expose the spinal cord. Surgical intervention was carried out to induce spinal cord transection, leading to hind limb paralysis in the rats. Rats in the control group underwent a sham surgery without SCI. Unlike the aneurysm clip compression model used in the dataset, our *in vivo* validation experiments employed a transection model, as this model offers greater uniformity of injury, clearer advantages for mechanistic studies, and higher feasibility for interventional experiments.

### 2.12 Tissue section preparation and immunofluorescence staining

Spinal cord tissues from normal rats and from rats at 1 and 2 weeks after SCI were used for immunofluorescence staining. At the designated time points, Sprague–Dawley rats were anesthetized by intraperitoneal injection of 2% pentobarbital sodium (30 mg/kg). After anesthesia, transcardial perfusion was performed through the left ventricle, first with normal saline and then with 4% paraformaldehyde for fixation. A spinal cord segment approximately 1.5 cm from the injury center was collected and post-fixed in 4% paraformaldehyde for 48 h. The tissues were then embedded in paraffin and sectioned at a thickness of 4 μm for subsequent analysis.

Paraffin sections were deparaffinized, subjected to antigen retrieval using EDTA buffer (pH 8.0; G1206, Servicebio, China), and washed with PBS. Sections were then blocked with 5% bovine serum albumin (BSA) for 30 min at room temperature to reduce nonspecific binding.

In the first immunofluorescence staining, the sections were incubated overnight at 4 °C with the primary antibody against IQGAP1 (1:200; GB114934, Servicebio, China). After washing with PBS, the sections were incubated with the corresponding secondary antibody (goat anti-rabbit IgG, CY3-conjugated, 1:300; GB21303, Servicebio, China) for 50 min at room temperature. The nuclei were counterstained with DAPI (G1012, Servicebio, China). After quenching autofluorescence, the sections were mounted and observed using a fluorescence microscope.

In the second immunofluorescence staining, each sample was subjected to five sets of double-staining experiments. In each experiment, IQGAP1 and DAPI were co-stained with one of the following cell-type markers:

GFAP(1:200; PGP055,Oasis biofarm), IBA1 (1:200; PGP049-01,Oasis biofarm), Olig2 (1:500; PGP040-02,Oasis biofarm), CD68 (1:4000;ab303565,Abcam), MAP2 (1:1000;AB5622,Merck).

After incubation with the corresponding primary and secondary antibodies, the nuclei were counterstained with DAPI. Images were captured using an Olympus fluorescence microscope.

To evaluate the colocalization of IQGAP1 with different cell markers, the Coloc2 plugin in ImageJ software (NIH, USA) was used for image analysis. Quantitative parameters, including Pearson’s correlation coefficient and Manders’ overlap coefficient, were calculated to assess the spatial relationship between IQGAP1 and each cellular marker.

### 2.13 Statistical analysis

Statistical analysis was performed using GraphPad Prism (version 9.02, GraphPad Software Inc., USA). Data are presented as mean ± SD. Unpaired Student’s t-test was used for comparison. P < 0.05 was considered statistically significant.

## 3 Result

### 3.1 Identification of DE-DRGs in SCI

**Figure 1:**
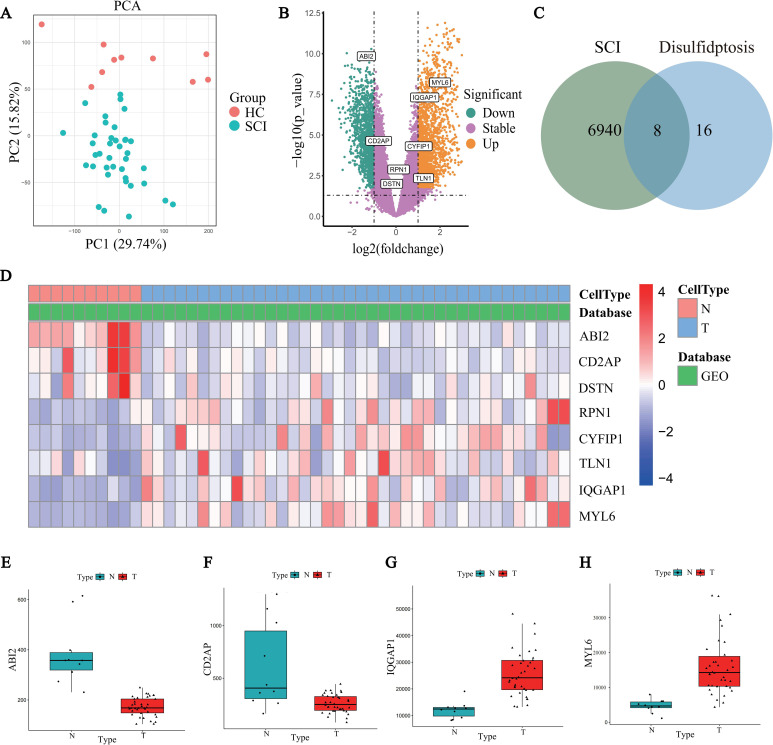
Identification and Expression Pattern Analysis of DE-DRGs in SCI. **(A)** PCA of the GSE151371 dataset, with different colors representing the SCI group (blue) and healthy control group (HC group, red). **(B)** Volcano plot showing the DEGs between the SCI and HC groups, with significantly upregulated and downregulated genes highlighted in orange and green, respectively. **(C)** Intersection analysis of 6948 SCI-related DEGs with 24 DRGs, identifying 8 DE-DRGs. **(D)** Heatmap displaying the expression levels of DE-DRGs in SCI samples, with colors ranging from red (high expression) to blue (low expression). **(E–H)** Box plots of representative DE-DRGs (ABI2, CD2AP, IQGAP1, and MYL6) showing significant expression differences between the SCI group (T) and HC group (N).

DEGs were analyzed using the GSE151371 dataset obtained from the GEO database. PCA was first performed to assess the overall expression trends of the samples. The results showed clear clustering differences between the SCI and healthy control groups, indicating good data quality and significant inter-group differences (**Figure 1A**). Next, differential expression analysis was performed using the limma package (adjusted *P*-value < 0.05), identifying a total of 6948 DEGs between the SCI and control groups (**Figure 1B**, **Supplementary Table 1**). These DEGs were intersected with the 24 previously reported DRGs, resulting in 8 DE-DRGs (**Figure 1C**, **Table 1**).

**Table 1:** The 8 DE-DRGs between the SCI samples and healthy samples.

Gene symbol	logFC	logCPM	*P*-value	Adj.*P*-value	Changes
ABI2	-1.155993695	3.740306523	3.73E-16	1.59E-13	down
MYL6	1.808966884	9.735372531	5.34E-10	1.67E-08	up
CD2AP	-1.237110231	4.348677392	3.6E-09	0.000000085	down
IQGAP1	1.123358364	10.48280015	3.66E-08	0.000000607	up
CYFIP1	0.890813427	5.233238347	0.00002	0.000134804	up
RPN1	0.583425683	7.165880728	0.000659249	0.00266589	up
DSTN	-0.573290433	3.665346004	0.002033639	0.006916869	down
TLN1	0.700642473	10.51496019	0.018920991	0.045988558	up

Additionally, a heatmap was used to display the expression levels of DE-DRGs, with a color gradient from red to blue representing gene expression levels from high to low. The heatmap clearly revealed significant expression differences of these genes between SCI patients and healthy controls (**Figure 1D**). Specifically, upregulated genes included MYL6, IQGAP1, CYFIP1, RPN1, and TLN1, while downregulated genes included ABI2, CD2AP, and DSTN. Expression analysis of representative DE-DRGs with adjusted *P*-value < 0.05 and |log_2_Fold Change| > 1 (ABI2, CD2AP, IQGAP1, and MYL6) showed significant differences between SCI patients (T) and healthy controls (N) (**Figures 1E–H**), further validating their potential key roles in the pathogenesis of SCI.

### 3.2 Evaluation of immune cell infiltration and drug prediction

**Figure 2:**
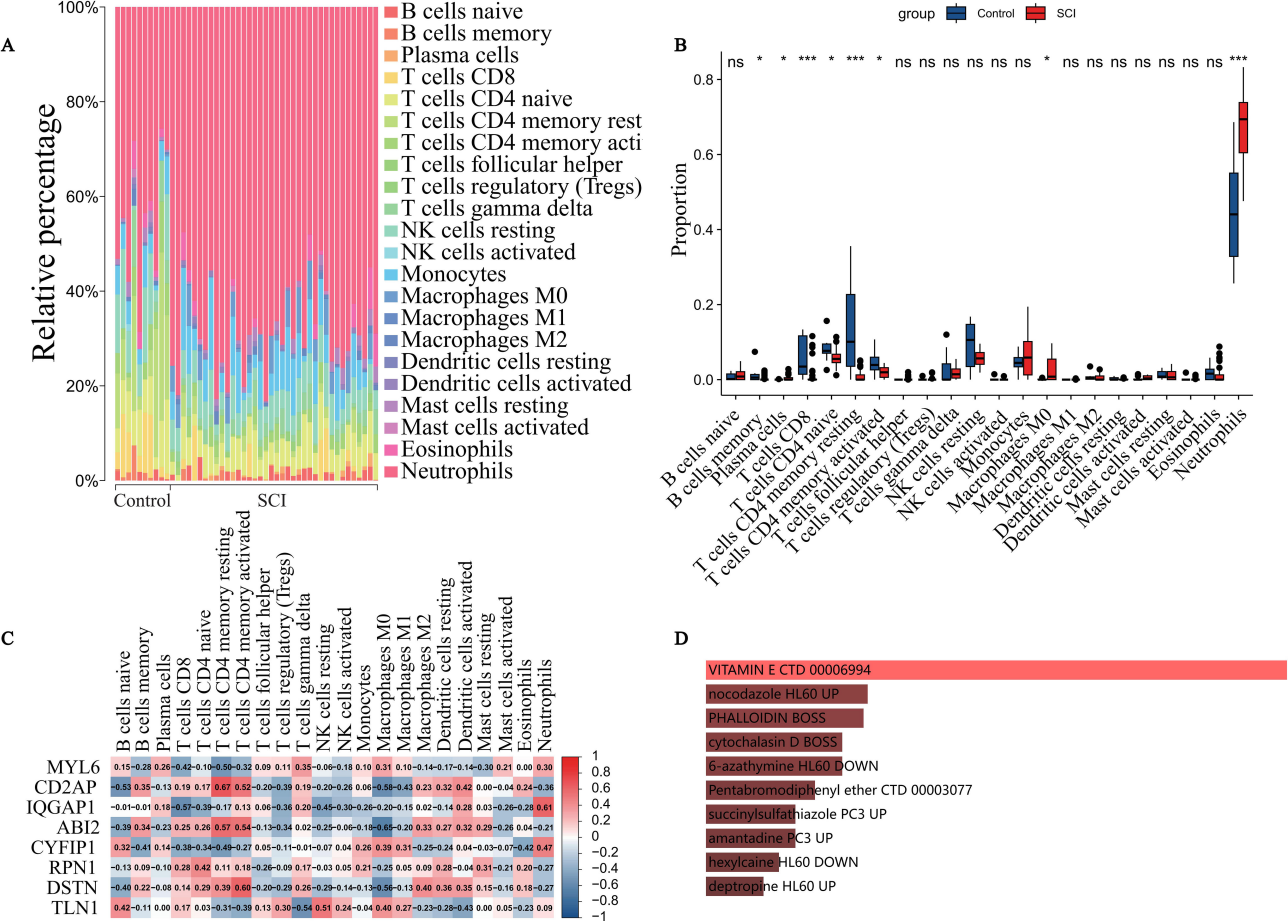
Immune Cell Infiltration Analysis and Drug Prediction in SCI. **(A)** Bar plot showing the relative proportions of 22 immune cell subtypes in each sample, as assessed by the CIBERSORT algorithm. **(B)** Comparison of immune cell infiltration proportions between the SCI and control groups, analyzed by the Wilcoxon rank-sum test (*P < 0.05, **P < 0.01, ***P < 0.001). **(C)** Heatmap of Spearman correlation between DE-DRGs and immune cell subtypes, with red indicating positive correlation and blue indicating negative correlation. **(D)** Bar plot of the top 10 predicted small molecules and compounds associated with DE-DRGs, with color intensity representing enrichment levels.

To investigate the role of the immune system in SCI, we evaluated the infiltration proportions of 22 immune cell subtypes in each sample using the CIBERSORT algorithm (**Figure 2A**). We then compared the differences in immune cell composition between the SCI and control groups (**Figure 2B**). The results showed significant differences in the proportions of 8 immune cell subtypes between the two groups (P < 0.05). Specifically, macrophages M0 and neutrophils were significantly increased in the SCI group, while the proportions of memory B cells, plasma cells, CD8+ T cells, CD4+ naive T cells, CD4+ memory resting T cells, and CD4+ memory activated T cells were significantly decreased. These changes suggest an immune activation imbalance and adaptive immune suppression in SCI, further supporting the potential role of DE-DRGs in immune regulation.The results revealed a distinct immune cell infiltration pattern in SCI samples. Correlation analysis between DE-DRGs and immune cells (**Figure 2C**) indicated a correlation between IQGAP1 expression and neutrophil proportion (R = 0.61), suggesting its potential involvement in SCI immune responses through the regulation of neutrophil recruitment or function.

### 3.3 Identification of candidate drugs and compounds

To explore potential drugs that may regulate DE-DRGs, we performed small molecule drug prediction (**Figure 2D**). Ten candidate compounds significantly associated with DE-DRGs were identified, ranked by their *P*-values (indicating their regulatory effects on the genes): vitamin E, nocodazole, phalloidin, cytochalasin D, 6-azathymine, pentabromodiphenyl ether, succinylsulfathiazole, amantadine, hexylcaine, and deptropine. Among these, vitamin E has a strong theoretical basis for application in SCI due to its antioxidant and neuroprotective effects.

### 3.4 Results of Mfuzz analysis

**Figure 3:**
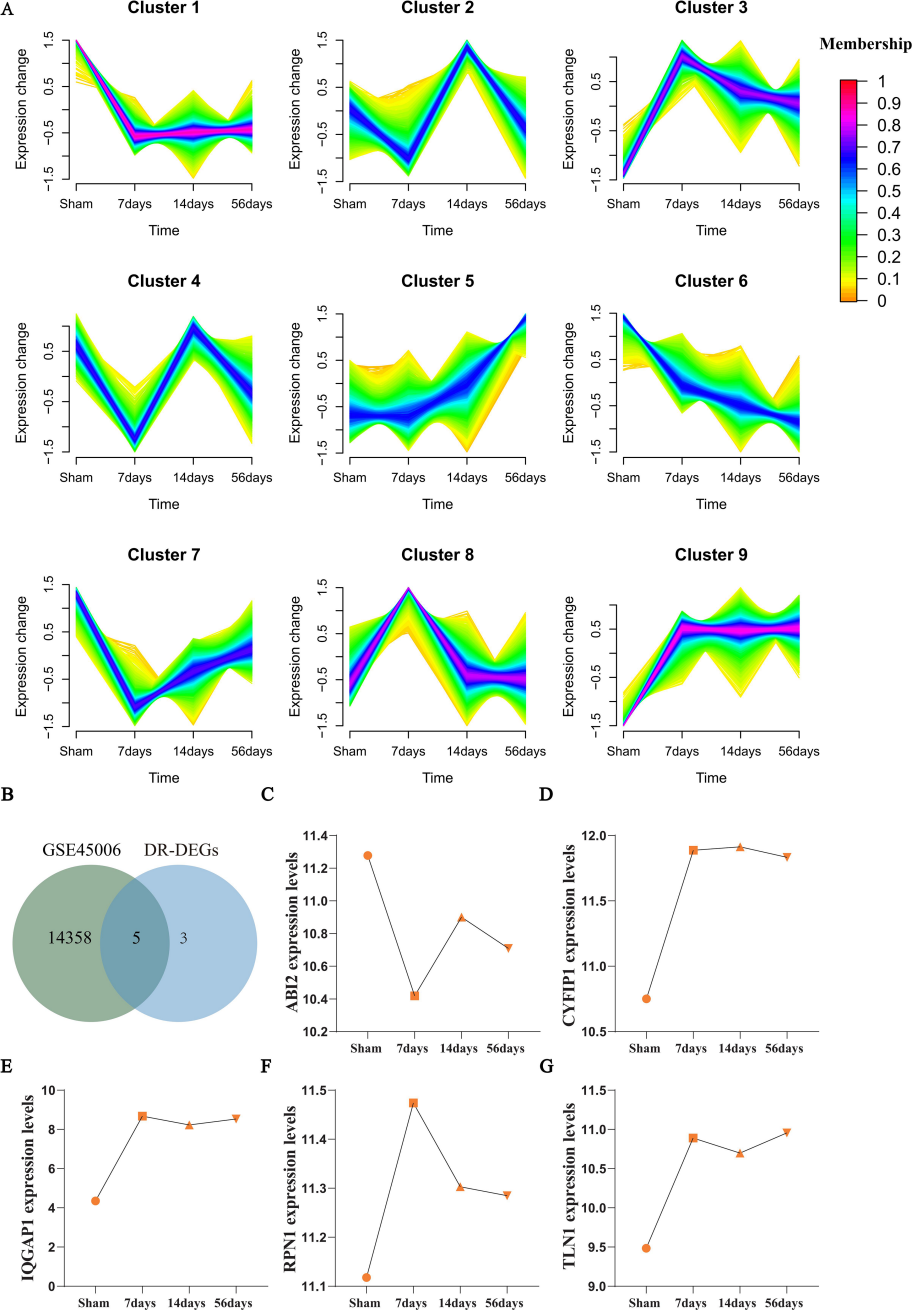
Results of Mfuzz Analysis. **(A)** The Mfuzz clustering analysis shows the expression patterns of 9 representative gene clusters at different stages of SCI, with colors representing membership values (red indicating high membership). **(B)** Venn diagram displaying the intersection of the GSE45006 gene set and DE-DRGs, identifying 5 overlapping key genes. **(C–G)** Expression trend plots of the 5 key genes (ABI2, CYFIP1, IQGAP1, RPN1, TLN1) at different time points, highlighting the early activation and sustained high expression of IQGAP1.

To explore the temporal expression patterns of DRGs following SCI, we performed time-series clustering analysis of gene expression dynamics using the Mfuzz algorithm based on the GSE45006 dataset. Nine representative gene expression clusters (Cluster 1–9) were identified (**Figure 3A**). Each subplot shows the expression trends of genes at four time points (Sham, 7 days, 14 days, 56 days), with color coding based on membership values, where red indicates the highest representation and yellow indicates the lowest membership.

We then performed an intersection analysis between the DEGs in this dataset and the DE-DRGs previously identified from the GSE151371 dataset, resulting in the selection of five key genes (ABI2, CYFIP1, IQGAP1, RPN1, and TLN1) (**Figure 3B**, **Supplementary Table 2**). The expression changes of these five genes across different time points were plotted (**Figures 3C–G**), and the results showed:

IQGAP1 (**Figure 3E**): As a core gene repeatedly confirmed in prior machine learning (RF, LASSO), PPI network, and ROC analyses, IQGAP1 was significantly upregulated on day 7 post-SCI (approximately doubled compared to Sham), and it maintained high expression at days 14 and 56. This trend suggests that IQGAP1 may first participate in the initiation of early inflammatory responses following SCI and continuously regulate various injury repair-related biological processes such as cytoskeletal remodeling, adhesion, and immune signaling, reinforcing its role as a core regulatory factor.

ABI2 (**Figure 3C**): Its expression level was significantly downregulated at day 7, suggesting its potential involvement in the suppression or remodeling process during the early stages of SCI.

CYFIP1 and TLN1 (**Figures 3D, G**): Both were rapidly upregulated at day 7 and maintained higher levels at days 14 and 56, possibly related to cell structure maintenance and immune activation.

RPN1 (**Figure 3F**): Its expression peaked at day 7 and gradually decreased, indicating its potential role in stress responses or protein transport regulation during the early stages of SCI.

In summary, the time-series dynamic expression analysis further validated IQGAP1’s continued involvement in the SCI pathological process, strengthening its credibility as a key disulfidptosis hub gene and a potential therapeutic target.

### 3.5 Identification of hub genes with diagnostic value through machine learning

**Figure 4:**
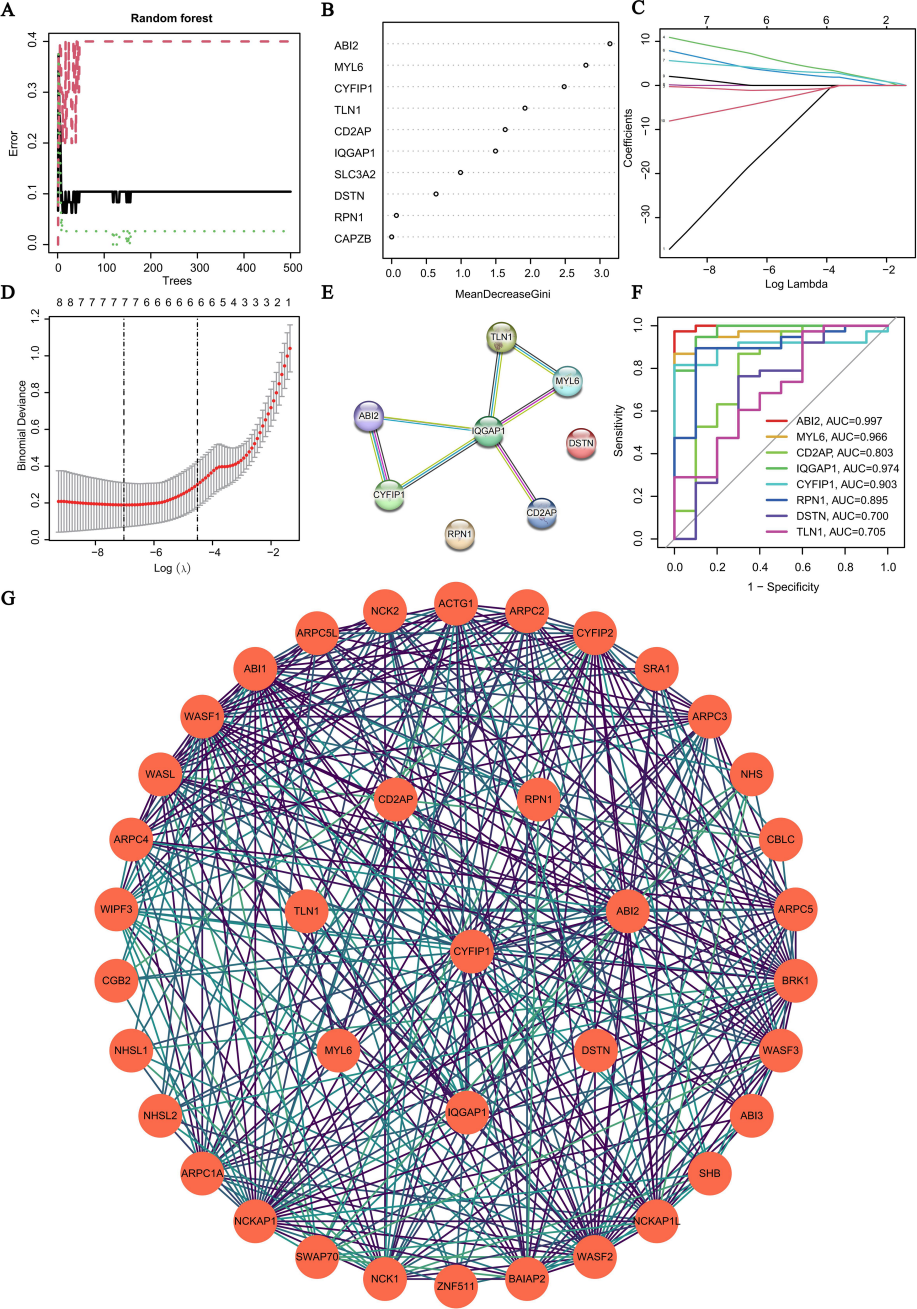
Identification of hub genes using machine learning and construction of PPI networks. **(A)** Error rate curve of the RF model. **(B)** Variable importance ranking based on the MeanDecreaseGini metric from the RF algorithm. **(C, D)** LASSO regression analysis: coefficient path plot and tenfold cross-validation to determine the optimal λ value. **(E)** STRING-based protein interaction network of the 8 DE-DRGs, with IQGAP1 located at the central position. **(F)** ROC curves evaluating the diagnostic performance of candidate genes in distinguishing SCI from healthy controls. **(G)** Extended PPI network comprising 38 nodes, showing IQGAP1 interacting with multiple signal regulation-related proteins.

To further identify key DE-DRGs with potential diagnostic value in SCI, this study utilized two mainstream machine learning methods. First, the RF algorithm was applied to assess the importance of each DE-DRG in classifying SCI and control groups, selecting 6 candidate genes with a MeanDecreaseGini value greater than 1, including ABI2, MYL6, CYFIP1, TLN1, CD2AP, and IQGAP1 (**Figures 4A, B**). These genes had high importance scores in the model, indicating their strong performance in distinguishing SCI from controls.

Then, the LASSO regression algorithm was used to further validate these results. The optimal λ value was selected through 10-fold cross-validation, and the LASSO model also identified the same 6 feature genes (**Figures 4C, D**), which were highly consistent with the RF algorithm results, reinforcing the stability and reliability of the feature selection.

Subsequently, we constructed a PPI network for the 8 DE-DRGs based on the STRING database, revealing a highly modular network structure (**Figure 4E**). IQGAP1 was located at the center of the network and exhibited significant interactions with other key genes, such as CYFIP1, ABI2, CD2AP, and TLN1, demonstrating its hub-like connectivity.

To assess the performance of these candidate genes in disease classification, we further constructed ROC curves and calculated the AUC values (**Figure 4F**). The results showed that ABI2 had an AUC of 0.997, indicating nearly perfect sensitivity and specificity, while IQGAP1 (AUC = 0.974), MYL6 (AUC = 0.966), and CYFIP1 (AUC = 0.903) also demonstrated high discriminatory power.

In summary, combining the results from RF and LASSO algorithms, the central position in the STRING network, and the excellent ROC curve performance, IQGAP1 was identified as a core regulatory gene closely associated with disulfidptosis in SCI, with significant potential for further mechanistic research and intervention.

### 3.6 Construction of PPI network

To further explore the broader protein interaction network and regulatory mechanisms of the candidate key genes, we extended the PPI network to include 38 nodes based on the STRING database (**Figure 4G**). Notably, IQGAP1, as a central hub protein, exhibited close interactions with several ARPC subfamily members (e.g., ARPC1A, ARPC2, ARPC3, ARPC4, ARPC5L), WASP family proteins (e.g., WASF1, WASF2, WASF3, WASL), and NCK pathway-related proteins (e.g., NCK1, NCK2). These interactions suggest that IQGAP1 may play a key regulatory role in SCI-related processes such as cytoskeletal remodeling, adhesion, and neuronal synapse reconstruction.

### 3.7 Molecular docking analysis

To assess the binding affinity between candidate compounds and the hub protein IQGAP1, molecular docking simulations were conducted. A Vina score lower than −5.0 kcal/mol generally indicates good binding activity, whereas a score below −7.0 kcal/mol suggests strong interactions. Among the ten screened small-molecule compounds with potential regulatory effects, succinylsulfathiazole, vitamin E, cytochalasin D, deptropine, hexylcaine, and nocodazole exhibited stable binding to IQGAP1, with binding energies all below −7.0 kcal/mol, indicating their potential to directly target IQGAP1 (**Table 2**).

**Table 2:** Binding energy for targets proteins with compounds.

Protein	Compound	Binding energy (kcal/mol)
IQGAP1	deptropine	-8.9
IQGAP1	cytochalasinD	-8.2
IQGAP1	hexylcaine	-8.1
IQGAP1	vitamin E	-8.0
IQGAP1	nocodazole	-7.9
IQGAP1	succinylsulfathiazole	-7.7
IQGAP1	amantadine	-6.5
IQGAP1	phalloidin	-6.5
IQGAP1	pentabromodiphenylether	-5.5
IQGAP1	6-azathymine	-5.1

**Figure 5:**
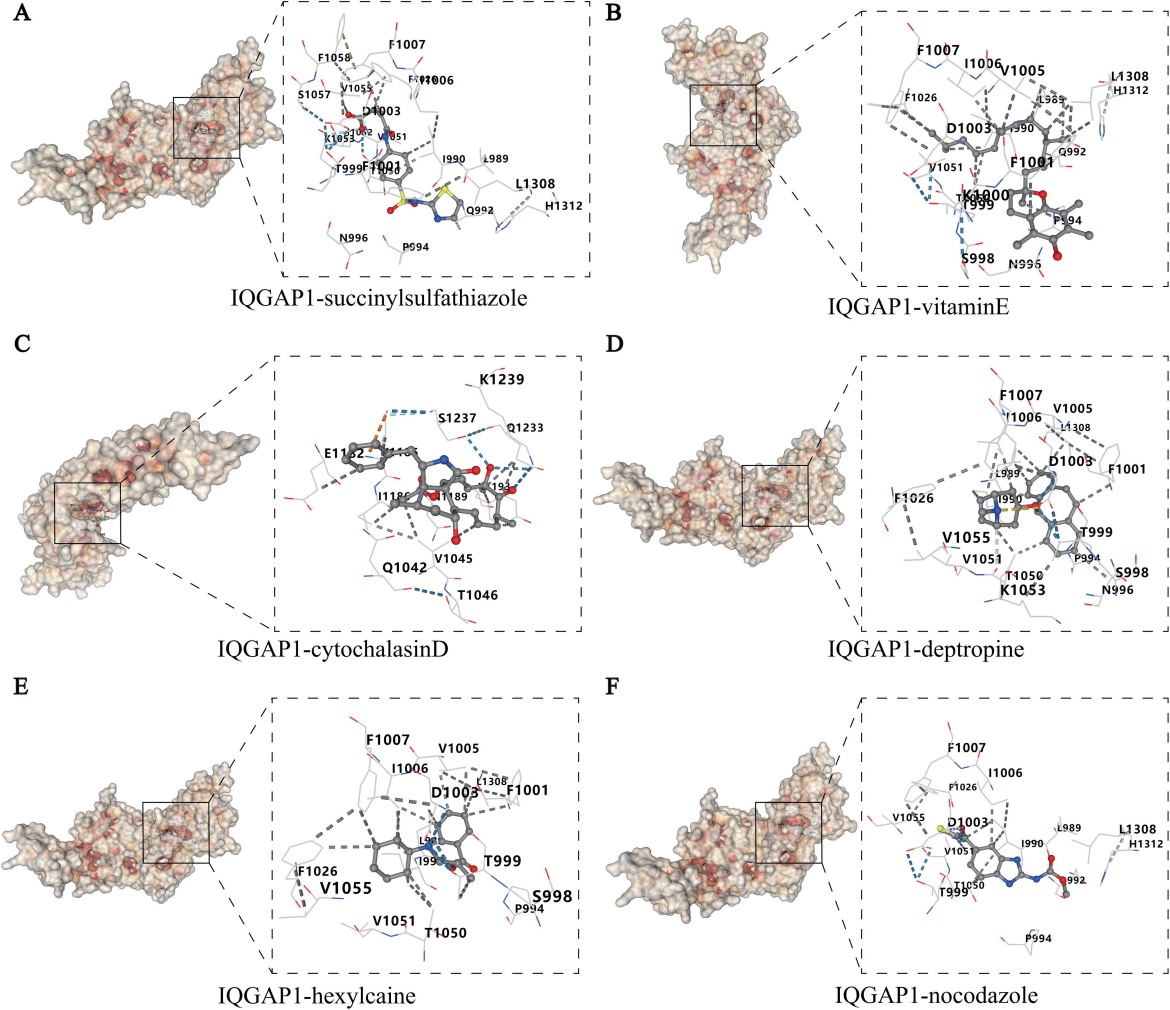
Molecular docking results of IQGAP1 with candidate small-molecule compounds. The left panels display the surface structures of IQGAP1, with hydrophobic regions highlighted in red. The right panels present enlarged 3D views of the binding pockets, illustrating the interaction patterns between the small-molecule ligands and IQGAP1 residues, including hydrogen bonds and hydrophobic contacts. Blue dashed lines represent hydrogen bonds, while gray lines indicate hydrophobic interactions. These results further confirm the favorable structural compatibility and binding affinity of the compounds with IQGAP1. **(A)** succinylsulfathiazole; **(B)** vitamin E; **(C)** cytochalasin D; **(D)** deptropine; **(E)** hexylcaine; **(F)** nocodazole.

**Figure 5** illustrates the docking conformations of the six compounds with IQGAP1, highlighting their binding sites within the IQGAP1 domains as well as the types of interactions involved, such as hydrogen bonds and hydrophobic contacts. Notably, several ligands formed stable non-covalent interactions with key amino acid residues of IQGAP1, including F1007, D1003, and V1055, further supporting its feasibility as a potential therapeutic target for disulfidptosis intervention in SCI. The docking conformations of the remaining four compounds are shown in **Supplementary Figure 1**.

### 3.8 GO and KEGG enrichment analyses

To further elucidate the potential biological functions of the core disulfidptosis-related gene in SCI, we first calculated the median expression level of IQGAP1 across all SCI samples. Based on this threshold, the 38 SCI samples were divided into high- and low-expression groups, with 19 samples above the median defined as the high-expression group and 19 samples below the median defined as the low-expression group. DEGs between the two groups were then subjected to GO and KEGG enrichment analyses. A total of 742 GO terms and 29 KEGG pathways (**Supplementary Table 3–6**) were identified (with thresholds set at *P* < 0.05 and FDR < 0.25), covering a wide range of cellular functions and pathological mechanisms.

**Figure 6:**
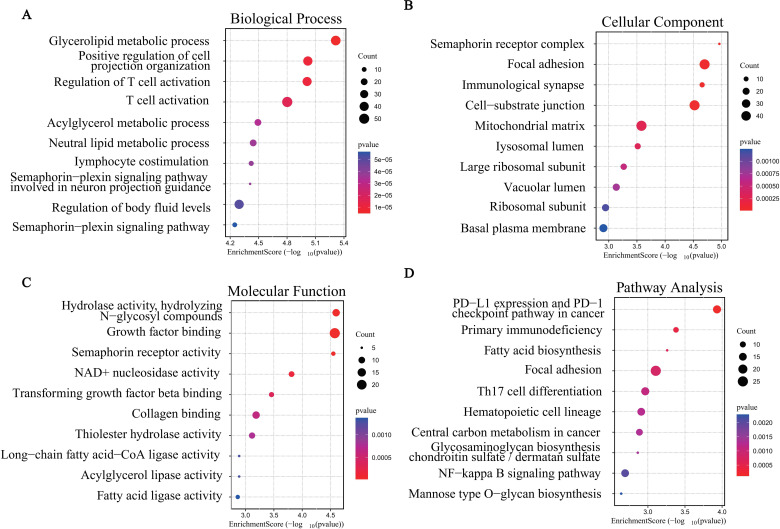
GO and KEGG enrichment analysis. Bubble plots showing the top 10 enrichment results of DE-ARGs in **(A)** BP, **(B)** CC, **(C)** MF, and **(D)** KEGG pathways.

In the GO analysis, biological process (BP) terms (**Figure 6A**, **Supplementary Figure 2A**) were significantly enriched in lipid metabolism (e.g., glycerolipid metabolic process, acylglycerol metabolic process), regulation of T cell activation, and neuronal projection organization (e.g., positive regulation of cell projection organization), suggesting that IQGAP1 may be involved in immune regulation and cytoskeletal remodeling. In terms of cellular component (CC) (**Figure 6B**, **Supplementary Figure 2B**), DEGs were mainly localized to the semaphorin receptor complex, focal adhesion, immune synapse, and mitochondrial matrix, indicating that IQGAP1 may participate in signal transduction and energy metabolism. Regarding molecular function (MF) (**Figure 6C**, **Supplementary Figure 2C**), genes were enriched in hydrolase activity, NAD+ nucleotidase activity, growth factor binding, and semaphorin receptor activity, supporting the multifunctional regulatory role of IQGAP1 in metabolism and signaling pathways.

In the KEGG pathway analysis (**Figure 6D**, **Supplementary Figure 2D**), significantly enriched pathways included PD-L1 expression and PD-1 checkpoint pathway, primary immunodeficiency, fatty acid biosynthesis, adherens junction, and Th17 cell differentiation. These pathways are closely associated with immune imbalance, metabolic disorders, and cell adhesion mechanisms in SCI, further underscoring the potential role of IQGAP1 as a key hub gene linking metabolic regulation and immune response.

### 3.9 GSVA and GSEA enrichment analysis in SCI

To further explore the potential biological differences at the pathway level between IQGAP1 high- and low-expression groups, we performed both GSVA and GSEA enrichment analyses on SCI samples.

**Figure 7:**
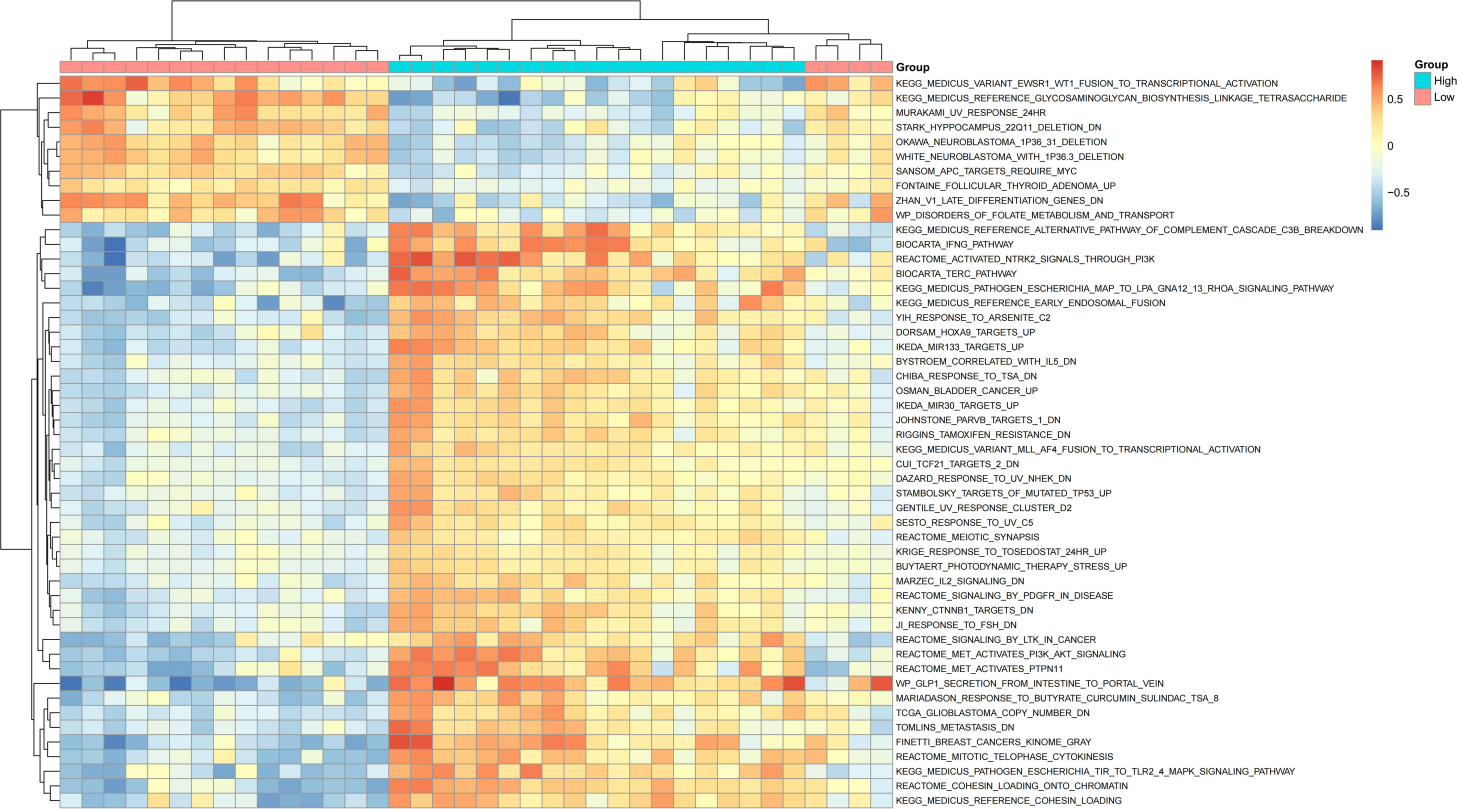
Heatmap of GSVA enrichment analysis showing pathway activity differences between high and low IQGAP1 expression groups.

In the GSVA analysis (**Supplementary Table 7**), pathway activity scores were calculated for each sample and visualized using a heatmap (**Figure 7**). The GSVA bar plot (**Supplementary Figure 3**) revealed that the high IQGAP1 expression group was significantly enriched in pathways associated with immune activation, cell cycle regulation, and DNA damage repair, including:

REACTOME_MET_ACTIVATES_PIK3_AKT_SIGNALING (PI3K-AKT signaling activation), MEDICUS_PATHOGEN_ESCHERICHIA_TIR_TO_TLR2_4_MAPK_SIGNALING_PATHWAY (MAPK-mediated pathogen recognition pathway), REACTOME_COHESIN_LOADING_ONTO_CHROMATIN (chromatin structure regulation), YIH_RESPONSE_TO_ARSENITE_C2, REACTOME_SIGNALING_BY_PDGF_IN_DISEASE, and OSMAN_BLADDER_CANCER_UP.

Conversely, the low-expression group showed significant enrichment in pathways related to metabolic downregulation, restricted development, and mutational responses, such as:

WP_DISORDERS_OF_FOLATE_METABOLISM_AND_TRANSPORT, MEDICUS_REFERENCE_GLYCOSAMINOGLYCAN_BIOSYNTHESIS, MURAKAMI_UV_RESPONSE_24HR.

These results suggest that samples with high IQGAP1 expression exhibit more active proliferative and immune responses, while low-expression samples may be associated with suppressed metabolism and developmental processes.

**Figure 8:**
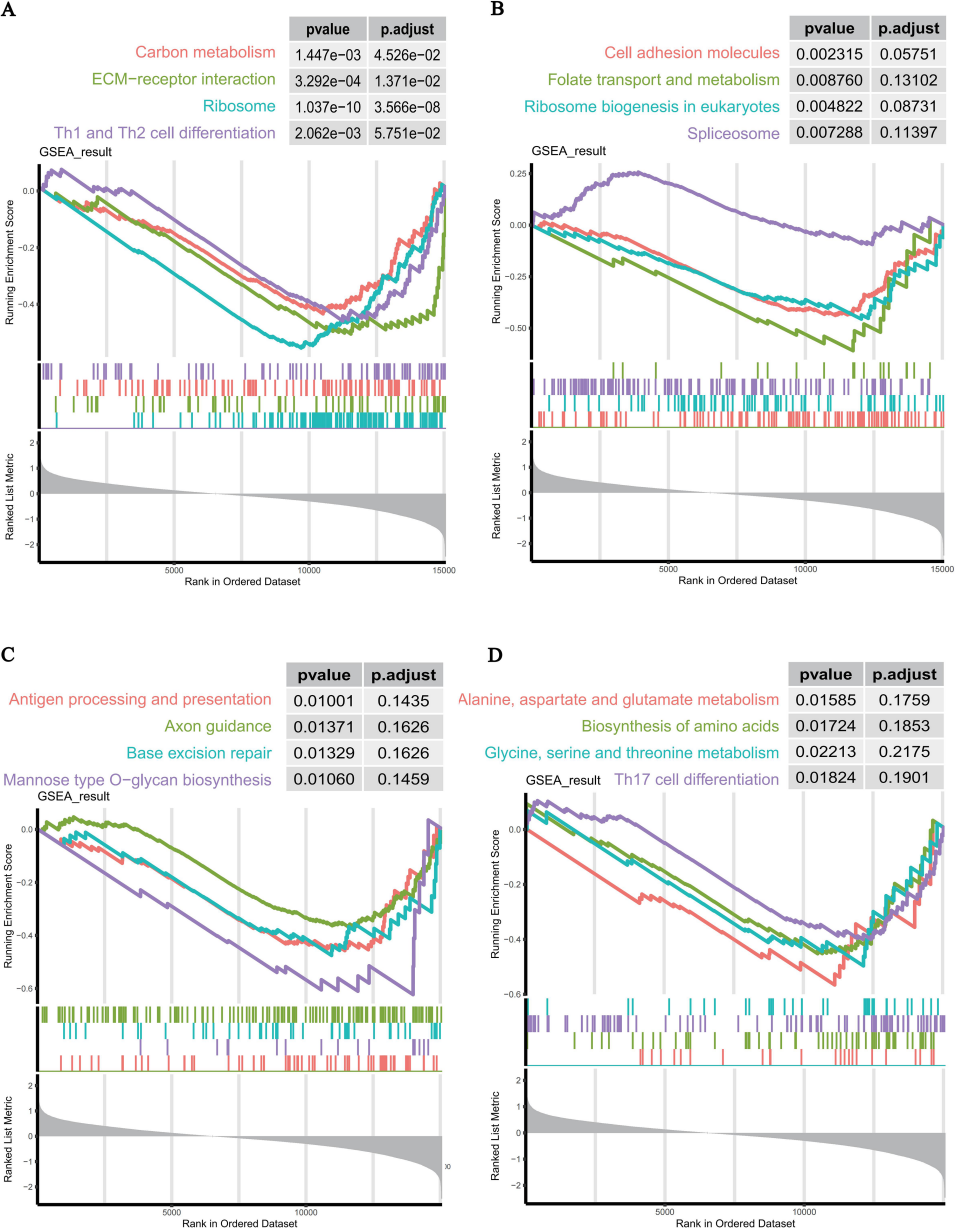
GSEA enrichment analysis results. **(A–D)** Representative GSEA plots showing key functional pathways significantly enriched in the high IQGAP1 expression group.

To further validate the functional pathways associated with IQGAP1 expression, GSEA enrichment analysis was performed (**Figures 8A–D**, **Supplementary Table 8**). Multiple SCI-related pathways were significantly enriched in the IQGAP1 high-expression group, including: ECM-receptor interaction, Carbon metabolism, Th1 and Th2 cell differentiation, Cell adhesion molecules, Ribosome biogenesis in eukaryotes, Spliceosome, Antigen processing and presentation, Base excision repair, Axon guidance, Biosynthesis of amino acids, Th17 cell differentiation, Glycine, serine and threonine metabolism.

Moreover, the density plot (**Supplementary Figure 4**) illustrated the distribution trends of GSVA scores across representative pathways, further supporting a positive correlation between IQGAP1 expression and multiple immune and metabolic signaling pathways.

### 3.10 Validation of IQGAP1 expression by immunofluorescence in rat SCI model

**Figure 9:**
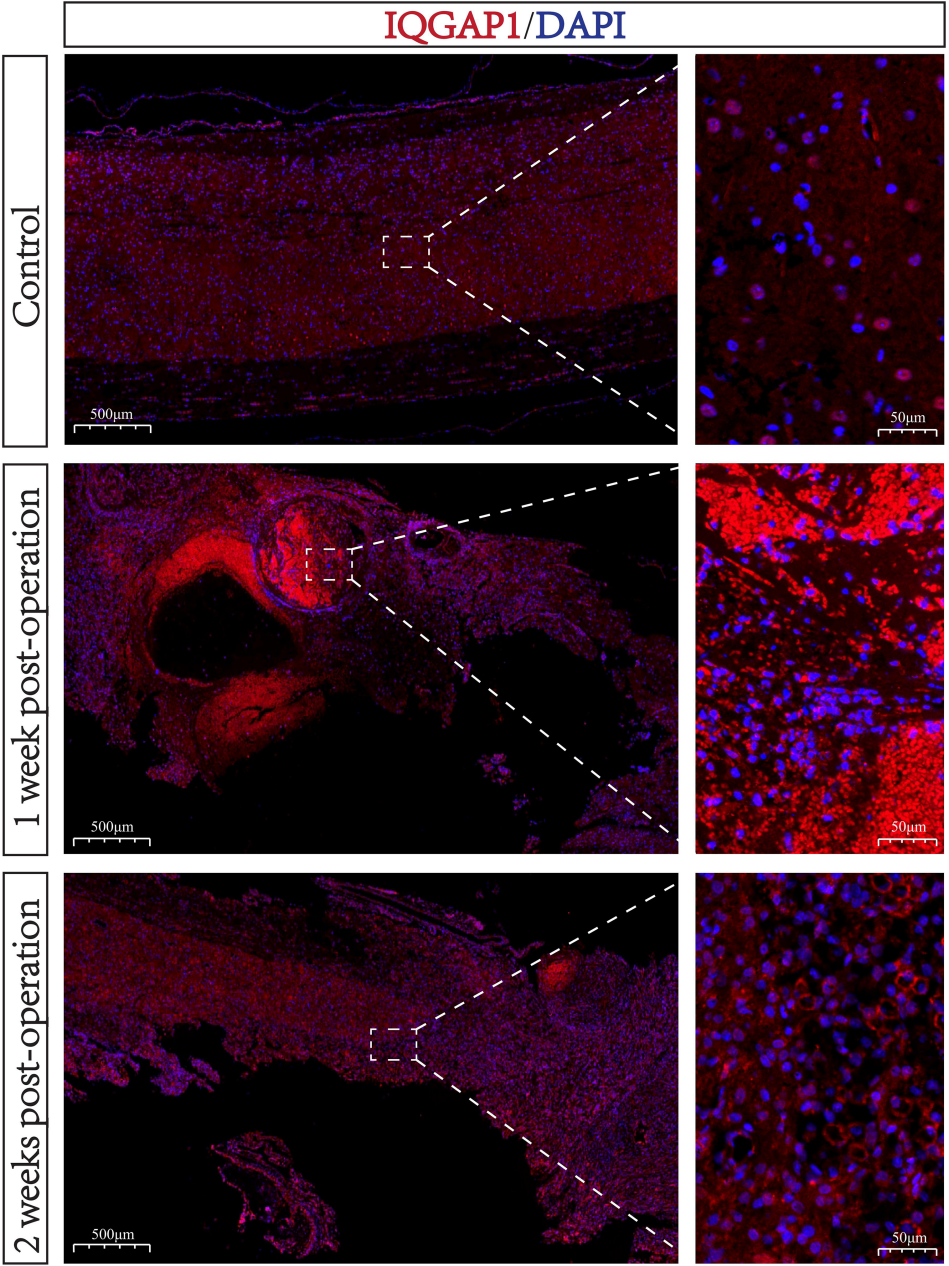
IQGAP1 expression in the lesion area at different time points following SCI. Representative immunofluorescence images showing IQGAP1 (red) and nuclei (DAPI, blue) staining in spinal cord sections from control rats (top), 1 week post-injury (middle), and 2 weeks post-injury (bottom). The right panels display magnified views of the boxed regions on the left. IQGAP1 expression was markedly increased in the injured area, peaking at 1 week post-injury and remaining elevated at 2 weeks, suggesting its potential involvement in SCI pathophysiology. Scale bars: left, 500 μm; right, 50 μm.

To validate the expression dynamics of IQGAP1 inSCI, we performed immunofluorescence staining on spinal cord tissue sections from a rat SCI model at one and two weeks post-injury (**Figure 9**). Compared with the uninjured control group, IQGAP1 (red) was markedly upregulated in the SCI region, with the strongest expression observed at one week post-injury. It was broadly distributed in the lesion core and surrounding areas, showing substantial overlap with DAPI-stained nuclei (blue). Although the expression slightly declined by the second week, it remained at a relatively high level (**Supplementary Figure 5**). This temporal expression pattern is consistent with our prior bioinformatic findings, suggesting that IQGAP1 not only participates in the early acute-phase response following SCI but may also continuously mediate cytoskeletal remodeling, inflammatory signaling, and cellular migration during the subacute phase, indicating its potential as a prognostic biomarker.

### 3.11 Temporal expression and colocalization changes of IQGAP1 in different cell types after spinal cord injury

**Figure 10:**
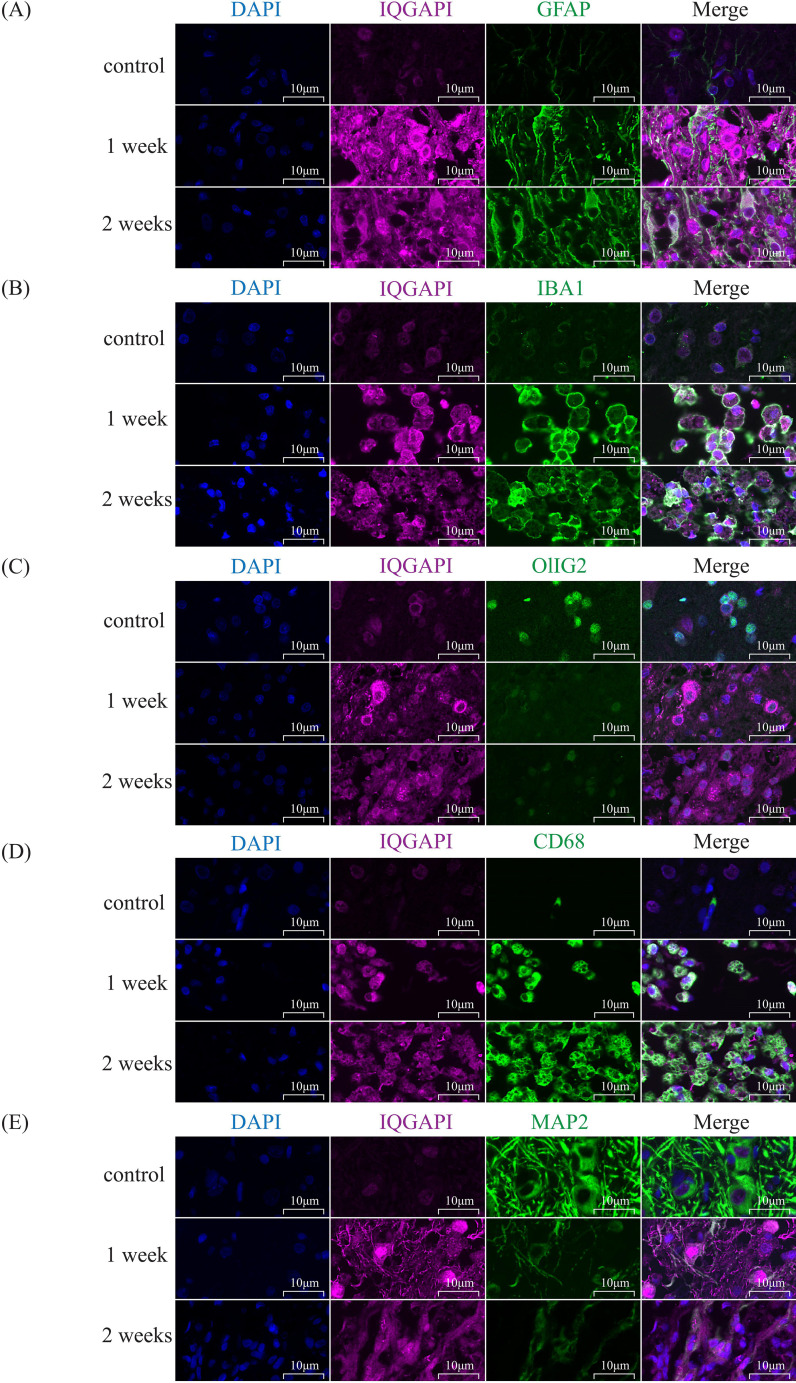
Colocalization of IQGAP1 with different cell-type markers after spinal cord injury. At 1 and 2 weeks post-SCI, spinal cord sections from control and injured rats were subjected to immunofluorescence staining. Sections were double-labeled with IQGAP1 (magenta) and cell-type markers (green), and nuclei were counterstained with DAPI (blue). Scale bar = 10 μm.

Immunofluorescence staining was employed to investigate the distribution of IQGAP1 in spinal cord tissue following spinal cord injury (SCI). As shown in **Figure 10**, IBA1 and CD68 signals were markedly increased at 1 and 2 weeks after injury and exhibited strong colocalization with IQGAP1, indicating that IQGAP1 is highly upregulated in activated microglia/macrophages during the inflammatory response. In contrast, although colocalization of GFAP with IQGAP1 was observed after SCI, its intensity was relatively weaker compared with that in IBA1^+^ and CD68^+^ cells. Likewise, the colocalization of IQGAP1 with oligodendrocytes (Olig2^+^) and neurons (MAP2^+^) was relatively weak, consistent with the loss of oligodendrocyte and neuronal structures after SCI (**Supplementary Table 9**). Taken together, these findings suggest that IQGAP1 expression is dynamically regulated in a cell type–specific manner after SCI, being preferentially upregulated in activated microglia/macrophages, while its association with astrocytes, oligodendrocytes, and neurons is comparatively limited.

## 4 Discussion

SCI is a severe CNS disorder characterized by complex secondary pathophysiological cascades involving apoptosis, inflammation, oxidative stress, and multiple forms of programmed cell death (2). Among the recently identified cell death modalities, disulfidptosis, a novel mechanism marked by cytoskeletal collapse and closely associated with redox imbalance has garnered increasing attention (13). However, the specific role of disulfidptosis in SCI has not yet been systematically investigated. In this study, based on transcriptome-based integrative analyses combined with experimental validation, we comprehensively identified key DRGs in subacute SCI at the genome-wide level for the first time. Moreover, we explored their potential immunological relevance, regulatory networks, and drug-targeting implications, aiming to provide novel insights and therapeutic targets for elucidating SCI pathogenesis.

Initially, 6,948 DEGs were identified from the GSE151371 dataset and intersected with 24 DRGs reported in previous literature. This analysis yielded eight DE-DRGs, including five upregulated genes (MYL6, IQGAP1, CYFIP1, RPN1, and TLN1) and three downregulated genes (ABI2, CD2AP, and DSTN). Based on integrative evidence from RF and LASSO feature selection, centrality within the STRING PPI network, and excellent diagnostic performance in ROC curve analysis, IQGAP1 was identified as a core regulatory gene closely associated with disulfidptosis in SCI. These findings highlight its potential as a valuable target for further mechanistic investigations and therapeutic intervention.

Further analysis of immune cell infiltration using the CIBERSORT algorithm revealed a positive correlation between IQGAP1 expression and neutrophil infiltration. In the SCI group, the proportions of neutrophils and M0 macrophages were significantly increased, while CD4^+^/CD8^+^ T cells, plasma cells, and memory B cells were markedly decreased. This pattern aligns with the complex pathological features of subacute SCI, characterized by concurrent inflammation amplification and immune suppression (24). Previous studies have demonstrated that IQGAP1, a multifunctional scaffolding protein involved in cytoskeletal remodeling, signal transduction, and inflammation regulation, plays pivotal roles in various inflammatory disorders and tumor immunity (25, 26). Through modulation of small GTPases such as Rac1 and Cdc42, as well as the MAPK signaling pathway, IQGAP1 influences immune cell chemotaxis, adhesion, and activation (27, 28).

In SCI, the initial mechanical trauma triggers an acute inflammatory response that rapidly recruits innate immune cells such as neutrophils and macrophages to the injury site (24). However, persistent inflammatory signaling may subsequently lead to T cell exhaustion and suppression of adaptive immunity (29). Our findings suggest that IQGAP1 may facilitate the recruitment and activation of neutrophils and M0 macrophages while concurrently inhibiting the infiltration of adaptive immune components such as CD4^+^ and CD8^+^ T cells. This implies that IQGAP1 may contribute to the establishment of inflammation-immunity imbalance within the SCI microenvironment. These results provide a theoretical basis for considering IQGAP1 as a potential target for modulating immune cell dynamics and mitigating secondary injury following SCI.

To explore the potential therapeutic agents targeting the identified DE-DRGs, we performed a drug enrichment analysis and identified a series of small-molecule compounds with regulatory potential, including vitamin E, nocodazole, phalloidin, and cytochalasin D. Among these, vitamin E, a classical lipid-soluble antioxidant, has been widely reported to exert neuroprotective effects in various CNS injury models by scavenging reactive oxygen species (ROS), inhibiting lipid peroxidation, and attenuating neuronal damage (30, 31). Its protective role is particularly evident during the secondary injury phase of SCI, where it mitigates oxidative stress, inflammation, and programmed cell death (32). Our molecular docking analysis further confirmed a strong binding affinity between vitamin E and IQGAP1 (binding energy < −7.0 kcal/mol), suggesting that vitamin E may participate in the regulation of disulfidptosis through modulation of IQGAP1-related signaling pathways. Considering that SCI secondary injury is often accompanied by oxidative stress, mitochondrial dysfunction, pro-inflammatory cytokine release, and cytoskeletal disintegration (24, 33), and given IQGAP1’s central role in cytoskeletal remodeling and signal integration, its function in mediating oxidative stress responses, cell adhesion, and inflammatory pathway activation is increasingly recognized (34). Therefore, vitamin E may not only attenuate secondary damage through its antioxidant properties but also exert a synergistic neuroprotective effect by targeting IQGAP1, modulating cytoskeletal dynamics and amplifying inflammation-related signaling.

Time-series expression analysis further confirmed a significant upregulation of IQGAP1 at day 7 post-SCI, which remained elevated at days 14 and 56. This suggests that IQGAP1 may play an important regulatory role during the subacute phase of SCI.

Results reported by R. Grillo et al. (35) are consistent with our **Figures 3C–G** at the overlapping time points. Importantly, Grillo et al. included an acute-phase comparison at days 0–3 that is not directly comparable to the “Sham” group in our study. Considered together, these datasets indicate that IQGAP1 upregulation begins in the acute phase (days 0–3) and remains elevated into the subacute phase (e.g., day 7), supporting a sustained post-SCI increase in IQGAP1 expression.

Previous studies have shown that IQGAP1 modulates the dynamic balance of F-actin in various cell types (36, 37). In epithelial cells, F-actin is linked to tight junctions and adherens junctions, contributing to the integrity of tissue barriers such as the blood–brain barrier and the blood–spinal cord barrier (38). Therefore, the sustained high expression of IQGAP1 during the subacute phase of SCI may help preserve the blood–spinal cord barrier or cellular polarity by regulating F-actin dynamics, thereby limiting the spread of inflammation.

After identifying IQGAP1 as a key gene, we divided the SCI samples into high- and low-expression groups (19 samples each) based on the median expression level of IQGAP1. On this basis, GSEA was performed to explore the potential signaling pathways associated with IQGAP1 expression, followed by GO, KEGG, and GSVA analyses to further elucidate the biological functions and mechanisms of the related genes.

GSEA results indicated that high IQGAP1 expression was strongly associated with pathways such as ECM–receptor interaction, axon guidance, cell adhesion, and T-helper cell differentiation, suggesting that IQGAP1 may influence post-injury tissue repair by modulating immune-inflammatory responses and cytoskeletal remodeling. Previous studies have demonstrated the role of IQGAP1 in regulating cytoskeletal stability and immune cell migration in inflammatory diseases (39, 40), which is consistent with our findings. Additionally, IQGAP1 is involved in downstream signaling of ECM proteins such as integrins and laminins, thereby regulating cell–matrix adhesion and traction forces (41). These interactions are critical for scar formation, vascular remodeling, and axon regeneration in SCI. Moreover, other members of the IQGAP family have been implicated in neuronal development, synaptic plasticity, and neurological disorders associated with altered dendritic spine density (42).

Further GO and KEGG enrichment analyses revealed that DEGs were significantly enriched in pathways related to T cell activation, immune synapse formation, lipid metabolism, and the PD-L1/PD-1 immune checkpoint signaling, supporting the notion that IQGAP1 may contribute to remodeling of the immune microenvironment following SCI via T cell–mediated immune regulation. Previous studies have confirmed that IQGAP1 localizes to the immune synapse in T cells, where it regulates F-actin remodeling, directional transport of signaling molecules, and immune synapse stability, which are functions essential for T cell activation (43). This mechanistic role explains the GO and KEGG enrichment of terms such as T cell activation and immune synapse, suggesting a potential role of IQGAP1 in the development of adaptive immune responses after SCI. Moreover, lipid metabolism plays a critical role in inflammatory signaling and membrane-associated signal transduction. GSVA analysis revealed that the high IQGAP1 expression group exhibited upregulation of pathways such as PI3K-AKT signaling, MAPK signaling, and DNA damage repair, while the low-expression group was mainly enriched in metabolism-suppressive pathways. These findings are consistent with previous reports that IQGAP1 activates PI3K-AKT and MAPK pathways (44). Specifically, IQGAP1-mediated activation of PI3K-AKT signaling contributes to endothelial cell migration and restoration of barrier function (45), and MAPK signaling is known to regulate macrophage M1 polarization to reduce secondary injury in SCI (46).

Taken together, these findings suggest that IQGAP1 may promote neural regeneration and tissue repair after SCI by activating immune response and DNA repair–related pathways. Additionally, IQGAP1-driven regulation of cytoskeletal stability and metabolic reprogramming may synergistically contribute to these effects. These results deepen our understanding of the biological role of IQGAP1 in secondary SCI pathology and provide a theoretical foundation for future targeted therapeutic strategies. Finally, using a rat model of SCI and immunofluorescence staining, we confirmed a marked upregulation of IQGAP1 at the lesion site, further supporting its role as a key regulatory factor in disulfidptosis during SCI. This experimental validation not only strengthens the reliability of our bioinformatics findings but also highlights IQGAP1 as a potential biomarker and therapeutic target for disulfidptosis-associated pathology.

In this study, we observed that IBA1 and CD68 signals were markedly enhanced at 1 and 2 weeks after SCI and showed strong colocalization with IQGAP1. This finding indicates that IQGAP1 is highly upregulated in activated microglia and infiltrating macrophages during the inflammatory response. Microglia are the resident immune cells of the central nervous system, while macrophages infiltrate from the periphery following disruption of the blood–spinal cord barrier. Both cell types play dual roles in SCI pathology: they contribute to the clearance of cellular debris but also exacerbate secondary injury through the release of pro-inflammatory cytokines (47, 48). The preferential upregulation of IQGAP1 in these immune cells suggests that it may participate in regulating cytoskeletal remodeling, migration, and pro-inflammatory signaling pathways, which underlie glial activation and immune infiltration. Previous studies have reported that IQGAP family proteins are involved in actin cytoskeleton organization and intracellular signaling, particularly in processes related to cell motility and adhesion (49, 50). Therefore, the strong colocalization of IQGAP1 with IBA1+ and CD68+ cells highlights its potential role in amplifying neuroinflammation after SCI. Further mechanistic studies are warranted to determine whether IQGAP1 directly regulates microglia/macrophage activation and polarization, and whether targeting this pathway could mitigate secondary injury and promote functional recovery.

Nevertheless, this study has several limitations. First, although the application of multiple bioinformatics approaches based on transcriptomic data enhances the robustness of our results, the expression data were derived from public databases and lack validation in large clinical cohorts. Second, the current understanding of disulfidptosis remains at an early stage, with incomplete characterization of its molecular markers, which may lead to mechanistic overlap or ambiguity. Further investigations using *in vivo* and *in vitro* models, including gene editing, protein co-localization, and functional inhibition experiments, are required to elucidate the precise role of IQGAP1 in mediating disulfidptosis and to evaluate its feasibility as a therapeutic target.

Third, we recognize a conceptual limitation in our study design. While our primary interest lies in the subacute phase of SCI, one of our key datasets (GSE151371) was derived from human peripheral blood samples collected within 48 hours after SCI, corresponding to the acute phase. Our rationale was to identify genes activated early and persisting into the subacute stage, which are more likely to drive secondary injury. To this end, we validated findings in the rat spinal cord dataset GSE45006, which spans both acute and subacute phases (1 day, 3 days, 1 week, 2 weeks, 8 weeks). Nonetheless, we acknowledge that the acute origin of GSE151371 may partially explain immunological patterns such as neutrophil predominance in CIBERSORT analysis, which are characteristic of the acute phase.

Fourth, we acknowledge the inherent limitations of combining data from different species (human vs. rat) and tissues (peripheral blood vs. spinal cord). Human and rat spinal cords differ in anatomy, immune composition, and injury/repair mechanisms, and peripheral blood leukocyte expression does not fully mirror the molecular landscape of injured spinal cord tissue. These discrepancies may affect the direct extrapolation of our findings to clinical settings. For example, immune responses in rats are often more acute and transient, whereas in humans they are more complex and persistent. Therefore, while cross-species and cross-tissue integration improves robustness by highlighting overlapping regulators, caution is needed when considering translational implications.

Finally, our selection of GSE45006 as the primary validation dataset also introduces a limitation. We chose GSE45006 because it provides a continuous time-series across acute, subacute, and early chronic phases (1 day to 8 weeks) within a single experimental framework, minimizing batch effects compared to merging multiple heterogeneous datasets. However, this meant that other available datasets used by Wang et al (19). were not included in our analysis. While our strategy reduced cross-platform variability and allowed us to focus on temporal dynamics, it may also limit the generalizability of our conclusions. In future studies, integrating additional datasets with larger sample sizes and complementary time points would further strengthen the robustness and translational value of our findings.

## 5 Conclusion

Through systematic bioinformatics analysis, this study is the first to investigate the potential role of disulfidptosis in subacute SCI. We screened and identified eight DE-DRGs closely related to disulfidptosis from the GEO database, among which IQGAP1 showed a significant upregulation trend and occupied a central hub position in the protein–protein interaction network, suggesting that it may play a key regulatory role in the secondary pathological process of SCI. Immune infiltration analysis, time-series clustering, and GSVA/GSEA enrichment further supported the important role of IQGAP1 in regulating inflammatory responses, cytoskeletal disassembly, and immune-metabolic remodeling. Drug prediction and molecular docking indicated that compounds such as cytochalasin D, deptropine, and nocodazole may target IQGAP1 to modulate the disulfidptosis process, providing potential candidate molecules for future therapeutic interventions. Validation by immunofluorescence confirmed the expression of IQGAP1 protein in SCI tissues, further strengthening the reliability of our findings. Moreover, five sets of double immunofluorescence staining demonstrated that IQGAP1 was predominantly upregulated in activated microglia/macrophages during the inflammatory response, thereby providing additional cellular-level evidence supporting its key role.

To summarize, this study is the first to systematically explore the potential involvement of disulfidptosis in subacute SCI and to identify key regulatory factors such as IQGAP1 along with candidate therapeutic compounds. Our findings provide new mechanistic insights into secondary SCI pathology and offer potential targets for the development of personalized intervention strategies.

## Data Availability

The original contributions presented in the study are included in the article/**Supplementary Material**. Further inquiries can be directed to the corresponding author.
