# Supplementary figures and images for: Identification of genes associated with disulfidptosis in the subacute phase of spinal cord injury and analysis of potential therapeutic targets
Source: Front Immunol. 2025 Oct 20;16:1642757. doi: 10.3389/fimmu.2025.1642757 (PMC12580332; doi:10.3389/fimmu.2025.1642757)

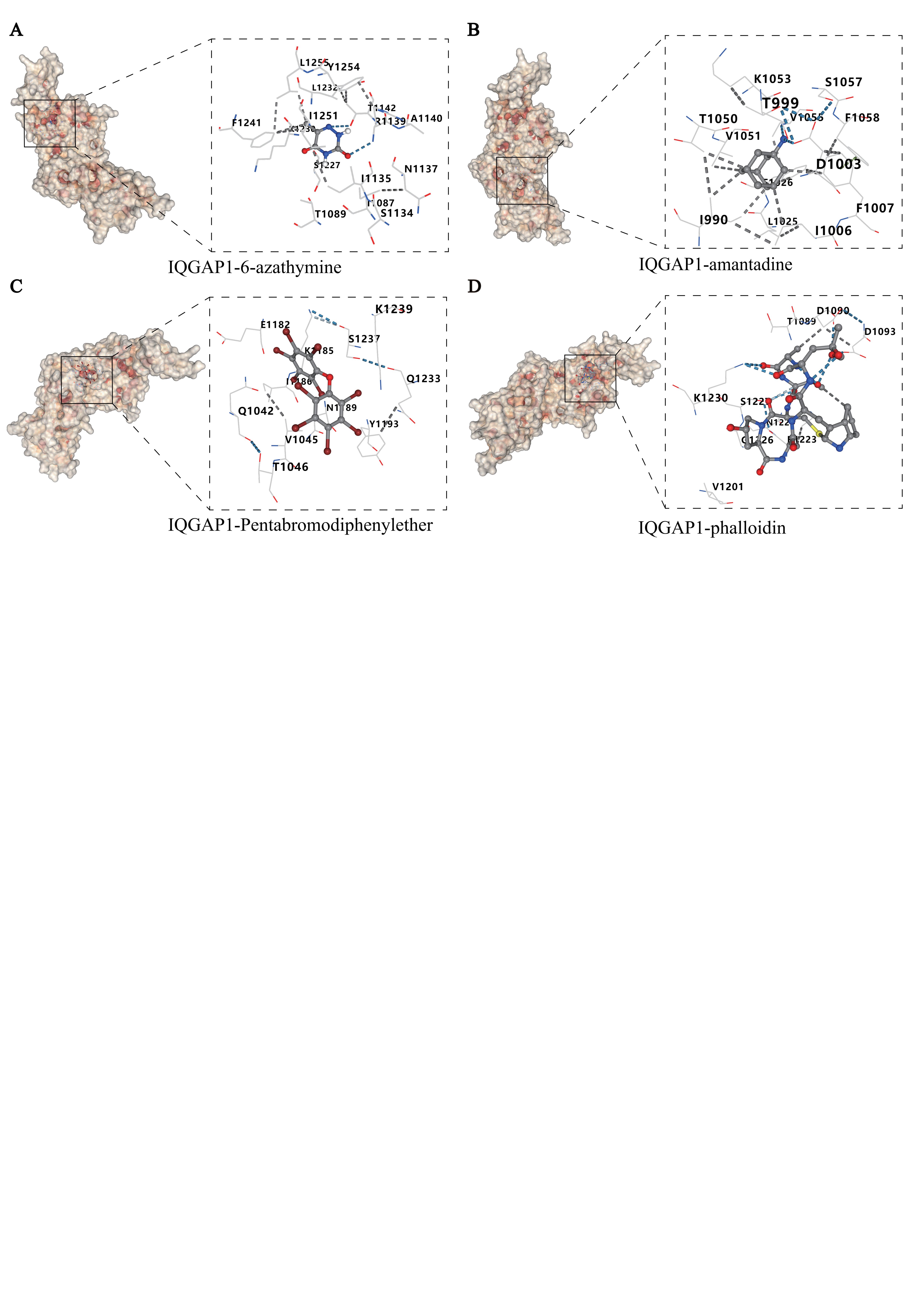

Supplement: Supplementary file 10 [file Image1.jpeg]

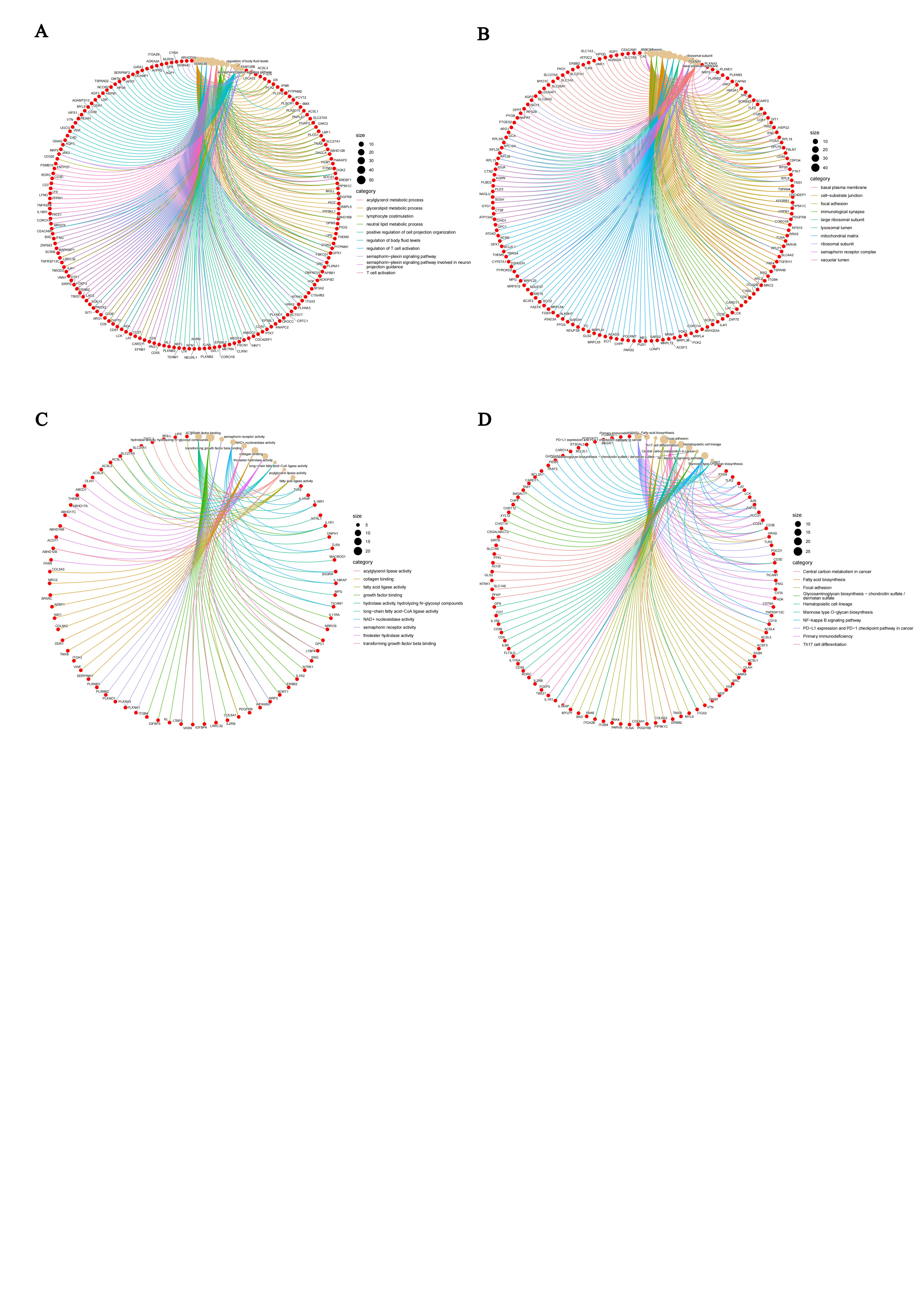

Supplement: Supplementary file 11 [file Image2.jpeg]

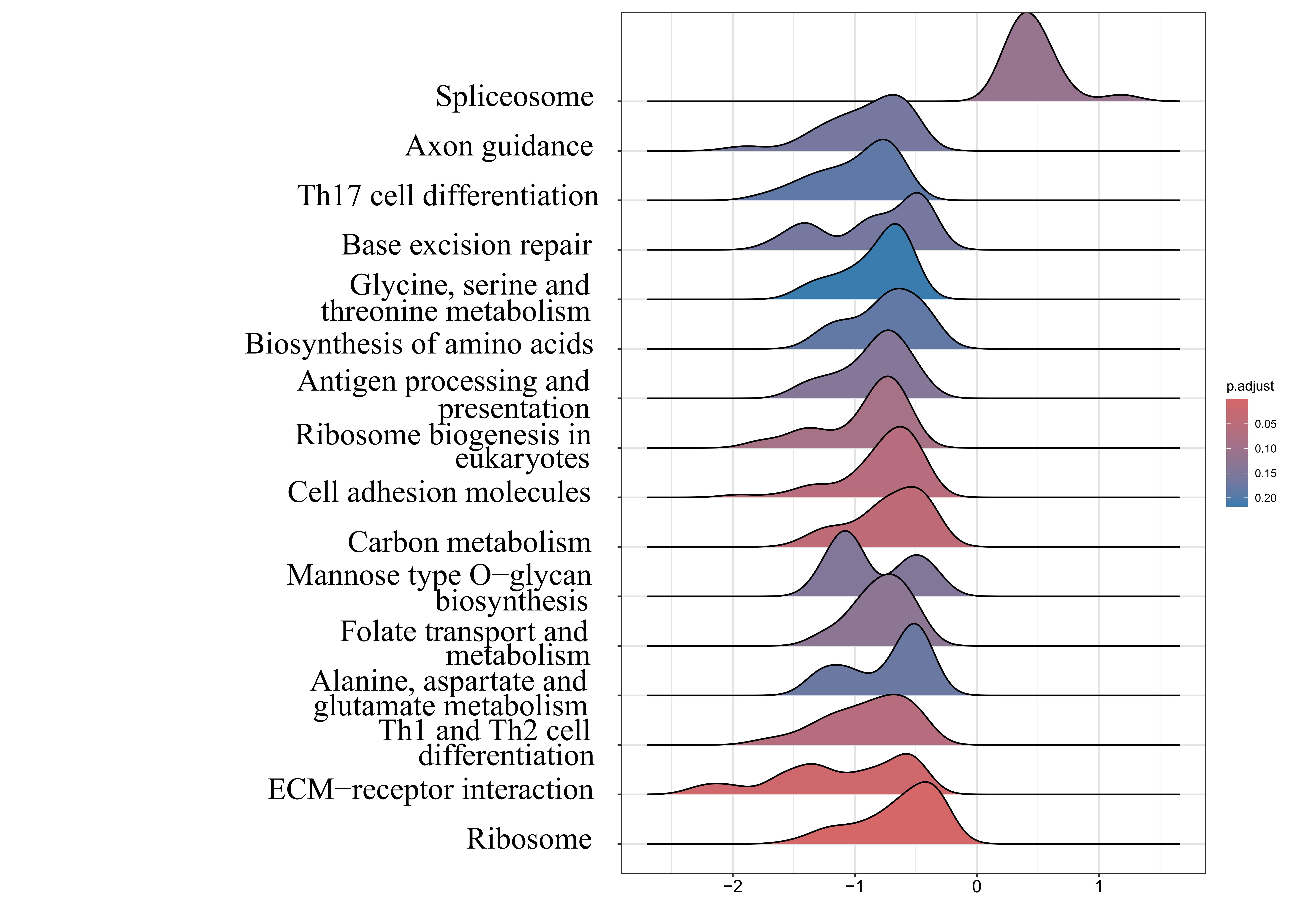

Supplement: Supplementary file 13 [file Image4.jpeg]

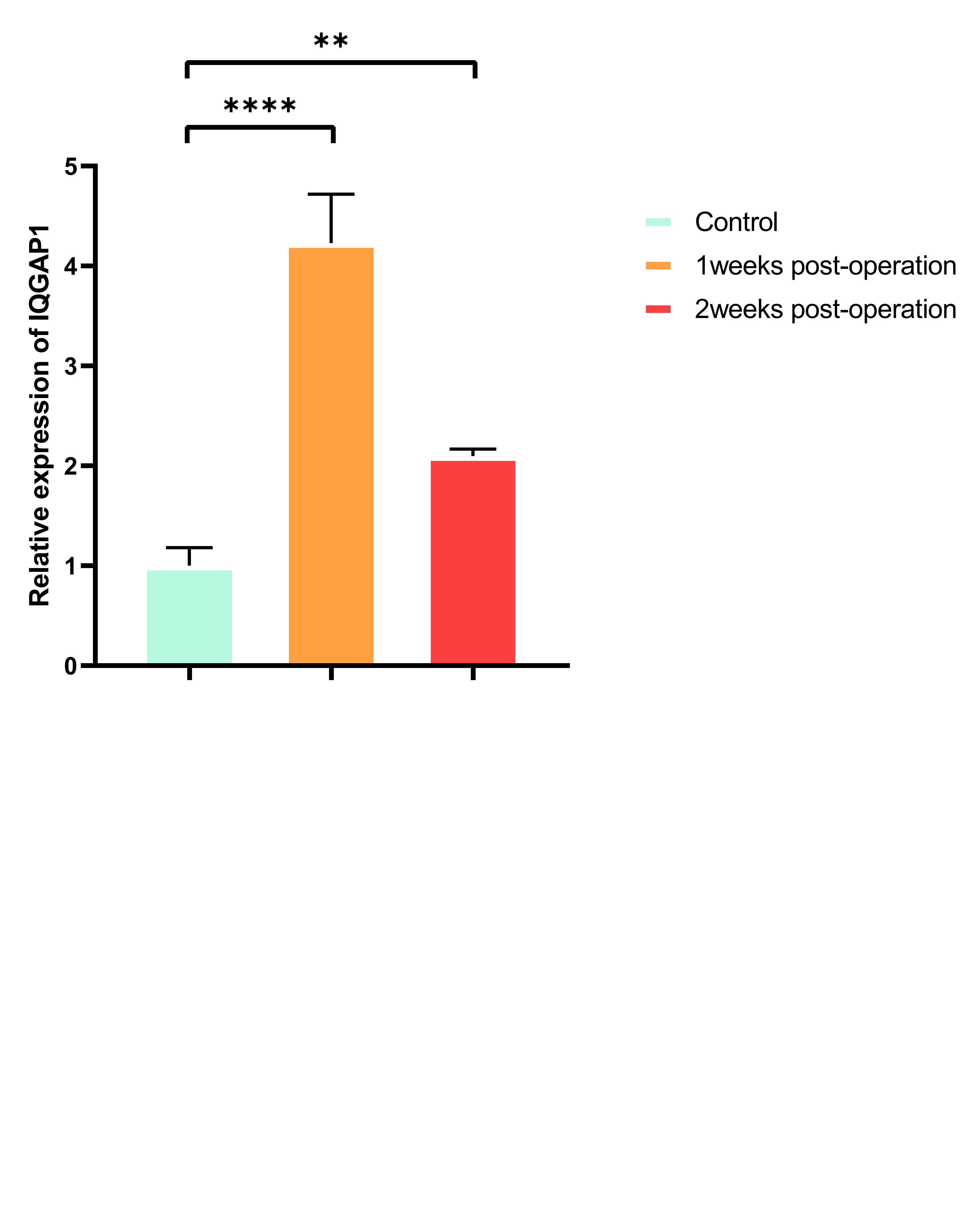

Supplement: Supplementary file 14 [file Image5.jpeg]
